# The Influence of β-1,3-1,6-Glucans on Rabies Vaccination Titers in Cats

**DOI:** 10.3390/vetsci7030118

**Published:** 2020-08-26

**Authors:** John Byrne, Darryn Knobel, Susan M. Moore, Stephanie Gatrell, Patrick Butaye

**Affiliations:** 1 Department of Biomedical Sciences, School of Veterinary Medicine, Ross University, Basseterre 00265, Saint Kitts and Nevis; dknobel@rossvet.edu.kn (D.K.); pbutaye@rossvet.edu.kn (P.B.); 2 Rabies Laboratory, College of Veterinary Medicine, Kansas State University, Manhattan, KS 66505, USA; smoore@vet.k-state.edu (S.M.M.); sgatrell@vet.k-state.edu (S.G.); 3 Bacteriology and Avian Diseases, Department of Pathology, Faculty of Veterinary Medicine, Ghent University, Ghent 9000, Belgium

**Keywords:** β-glucans, RFFIT, rabies, vaccine, antibody, titer, immunostimulant

## Abstract

β-glucans have been shown to stimulate the immune system in several animal species. The aim of this study was to evaluate the immune stimulation capacity of a fully formulated diet with β-1,3-1,6-glucans in cats, by assessing the rabies antibody titer after vaccination. Thirty-five healthy cats were recruited. The cats were placed into two groups and fed a standard diet in accordance with body weight. One group had the β-glucans incorporated into the diet; the other group served as the control group. After two weeks of dietary adjustment; the rabies vaccine (Imrab^®^ 3 TF; Merial) was administered on days 0 and 21. Blood samples were taken on days 0, 21, and 42. Titers were determined with the rapid fluorescent foci inhibition test (RFFIT). Titers at days 21 and 42 were compared between the two groups in a linear mixed effects model. This study showed that the animals receiving the non-supplemented feed had higher post-vaccination rabies antibody titers. This indicates that, in contrast to other animal species, the β-glucan supplemented diet did not have the expected positive effect on the rabies antibody titers in cats.

## 1. Introduction

Prebiotics are food ingredients that exert beneficial effects by altering the metabolism in the intestinal tract [1]. β-1,3-1,6-glucans, derived from the yeast cell wall of *Saccharomyces cerevisiae* fall into this category. When extracted from the cell wall of fungi, these polysaccharides can also work as immunomodulators, by boosting the host’s immune response to antigens in many immuno-therapies [2]. β-glucans have important effects on the immune response. Working through the gut-associated lymphoid tissue (GALT), β-glucans will bind to the transmembrane protein receptors, TLR2/TLR6 (Toll-like receptors), and dectin-1 on dendritic cells, follicular dendritic cells, macrophages, and even B-cells [3,4]. β-1,3-1,6-glucans enhance innate immune cellular abundance and function. Through the primary stimulus of β-1,3-1,6-glucans, there will be an increased abundance of antigen-specific lymphocytes that result in an increased innate immune response, as well as an increase in humoral and cellular immune response after vaccination [5,6].

Previous studies in dogs [7], mice [8], and pigs [9,10] have demonstrated that β-1,3-1,6-glucans produce an increase in the immunological response to rabies vaccination. There are no such data available for cats, therefore, we investigated the effect of β-1,3-1,6-glucans, added to a balanced cat diet, on rabies antibody titers following rabies vaccination.

## 2. Materials and Methods

### 2.1. Animals

The animal trial was approved by the RUSVM IACUC under number 19.05.18.

Thirty-six cats were recruited from owners, mainly veterinary students, who signed an informed consent document. Cats underwent a health check before inclusion and were housed with their owners. The exclusion criteria were as follows: kittens younger than 3 months, visibly old cats/cats older than 10 years, cats with poor body condition, and seropositive rabies antibody tested cats. Cats were to be housed inside for the entire study to increase control over the diet. One cat was withdrawn from the study at the owner’s request.

### 2.2. Study Design

This was an owner- and investigator-blinded study. The vaccination date was taken as day 0. On day 21, a 1 mL blood sample was collected for determination of the presence of rabies antibodies. The BioPro ELISA test was used as the initial screening test (day 21 sample) to demonstrate that the animals were serologically negative for rabies antibodies. The BioPro ELISA test was executed according to the instructions of the manufacturer [11]. Cats’ age, sex, and weight were also determined on day-21. Prior to day 14, the rabies antibody negative animals were allocated to two groups. Subjects in Group A were given a diet without β-1,3-1,6-glucans (control group) and subjects in Group B were given the same diet supplemented with β-1,3-1,6-glucans (test group). The cats were assigned to a group based on a similar distribution of age and sex. The change in diet for both groups began fourteen days before vaccination. On day 0, a 3 mL blood sample was taken for baseline rabies virus neutralizing antibody (RVNA) titers, after which the animals were vaccinated with rabies vaccine (Imrab^®^ 3 TF, Merial, Lyon, France). All blood samples were refrigerated for 24 h, then centrifuged at 2500 rpm for 10 min, after which serum was collected, placed into pre-labeled tubes, and stored at −80 °C. On day 21, a new 3 mL blood sample was taken for RVNA titer determination, after which the animals received a booster vaccination to increase the titers, to better see an immunological response. The trial ended on day 42 with a final 3 mL blood draw for endpoint RVNA titers. RVNA titers in sera at days 0, 21 and 42 were determined by the rapid fluorescent focus inhibition test (RFFIT), performed at the Kansas State University Rabies Laboratory [12].

### 2.3. Statistical Analysis

We used R [13] and the *lme4* package [14] to perform a linear mixed effects analysis of the relationship between post-vaccination RVNA titers and diet. As fixed effects, we entered diet (group A vs. group B) and time (days 21 and 42) into the model. As random effects, we had intercepts for subjects. A visual inspection of residual plots did not reveal any obvious deviations from homoscedasticity or normality. *p*-Values were obtained by likelihood ratio tests of the full model with the effect in question against the model without the effect in question.

## 3. Results

Thirty-six cats were enrolled in this study. Thirty-five cats were screened at day 21 with the BioPro ELISA Rabies AB+ Kit; one cat was withdrawn from the trial prior to initial ELISA test at the owner’s request. Group A was comprised of 18 cats (9 males and 9 females), while Group B contained 17 cats (9 males and 8 females), as shown in Table 1. The average weight for Groups A and B were 3.75 kg and 3.72 kg, respectively. The average ages of Groups A and B were 13.2 months and 14.2 months, respectively.

**Figure 1:**
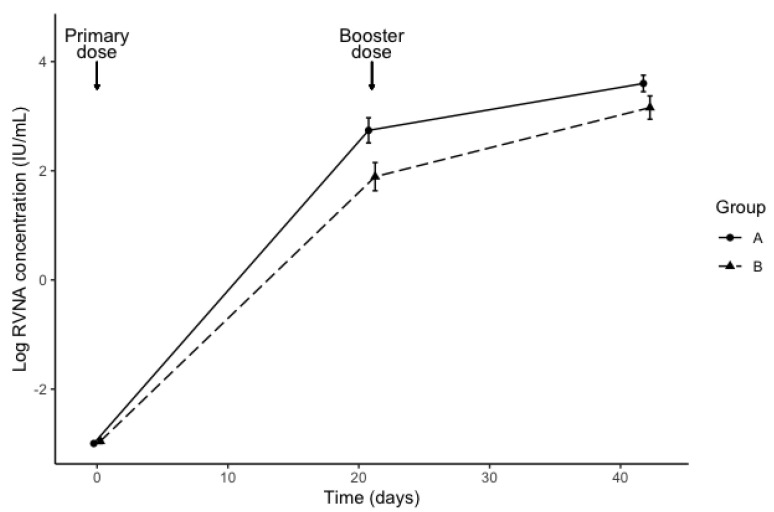
Rabies antibody levels (mean ± standard error) determined by the rapid fluorescent foci inhibition test (RFFIT) at 0, 21 and 42 days after rabies vaccination in previously-unvaccinated cats fed a control diet (Group A) or an identical diet supplemented with β-1,3-1,6-glucans (Group B), for 14 days prior to vaccination.

All 35 of the cats tested negative for rabies antibodies, and thus all were eligible for the study. RVNA titer results taken from sera at day 0 showed that all cats had titers ≤0.1 IU/mL. The RVNA levels ranged from 13.10–131 IU/mL in group A, and from 3.3–131 IU/mL in group B. The geometric mean concentration (± standard error) at day 21 was 15.5 ± 2.7 IU/mL for group A and 6.6 ± 2.9 IU/mL for group B. The geometric mean concentration at day 42 was 36.6 ± 1.9 IU/mL for group A and 23.5 ± 2.4 IU/mL for group B. Figure 1 shows the log-transformed RVNA levels by diet group and time. The interaction between diet and time was not statistically significant (ꭓ^2^ (1) = 1.50, *p* = 0.22). Diet affected RVNA levels (ꭓ^2^ (1) = 6.24, *p* = 0.012), with levels of cats in Group B lower by an estimated 1.9 IU/mL ± 1.3. In an exploratory analysis, the effect of sex was not statistically significant (ꭓ^2^ (1) = 1.09, *p* = 0.30). All cats (irrespective of diet) showed an adequate serological response to rabies vaccine.

## 4. Discussion

Studies in dogs [7], mice [8], pigs [9,10], horses [15] and various other species, including fish [16,17] and invertebrates [18], have demonstrated that β-1,3-1,6-glucans, derived from the yeast cell wall of *Saccharomyces cerevisiae*, produce an increase in the immunological response to foreign antigens. In our study, although both groups of cats developed an adequate neutralizing antibody response to a primary and booster injection of the rabies vaccine, the response was lower in cats that were fed a diet supplemented with β-1,3-1,6-glucans, compared with cats that were fed an un-supplemented diet, contrary to what was seen in other species.

A major pathway through which β-1,3-1,6-glucans stimulate and prime the immune system is through the dectin-1 surface protein in conjunction with TLR 2/TLR 6. This pathway has been shown to cause a change in CD45+, CD8+, and CD4+ T-cells, in the mesenteric lymph nodes, as well as the Peyer’s patches [19]. The stimulation by β-glucans has been shown to have a significant increase in the production of IL-12, IL-6, and TNF-α [20]. These pro-inflammatory cytokines aid in the production and proliferation of Th-1, Th-2, NK-cells, B-cells, and the production of immunoglobulins by plasma cells [21]. Although in most species these pathways support the immune stimulation, it did not support the same effect in cats. Studies in dogs have shown that both the cell-mediated and humoral responses of the innate and adaptive immune system were beneficially affected by the introduction of β-glucans [7,22]. While dogs and cats share similar immunological pathways and mechanisms [23], there is no clear explanation as to why there was not a similar response in cats.

One clear difference between the immunology of cats and dogs is the presence of the genes encoding the major histocompatibility complex (MHC). The genetic makeup of dogs’ MHC II, unlike that of cats, has been shown to have all three of the genes enabling proper antigen presentation. The dog leukocyte antigen complex (DLA) genes for MHC II are made up of DLA-DP-DQ-DR; in contrast, the feline leukocyte antigen complex (FLA), which is made up of FLA-DP-DR, shows a severe decrease in the function of the DP, which may explain an increased susceptibility to autoimmune disorders in felines [24]. It could be that the already naturally hindered pathway taken from MHC II to the CD4+ cells to eventually induce an antibody response in cats, may be interrupted by the introduction of β-1,3-1,6-glucans. With the increase of IL-12 and TNFα, β-glucans may cause an epigenetic shift that favors the production of proinflammatory cytokines as well as natural killer cells, as opposed to other species showing increases in antibody response. Further research looking at the cytokines produced, and the level at which they are produced, will help explain what pathways are favored depending on the presence or absence of β-1,3-1,6-glucans in the diet of cats.

Our knowledge of the intestinal bacterial microbiome of both dogs and cats is evolving rapidly, and it has been shown that the intestinal microbiome of cats is much more diverse than the microbial communities in the intestine of dogs [25,26]. The diversity between cats is however smaller than that of the diversity between dogs. Though both species are carnivores, capable of eating meat and plants, the narrow food requirements of cats as obligate carnivores require more precision in the formulation of its diets, relative to the more omnivorous dogs [27]. It was postulated that *Saccharomyces* is not an indigenous organism in any species’ intestinal microbiota, but rather, is found only after nutritional consumption [28]. *Saccharomyces* species are, however, found in large quantities in the intestinal microbiome of cats, and this may be an indication as to why the β-1,3-1,6-glucans, derived from the yeast cell wall of *Saccharomyces cerevisiae*, have had a different effect compared to what is seen in other animal species. The supplementation with extra β-glucans, resulting in even higher levels of β-glucans, might be a reason for why the β-1,3-1,6-glucan supplemented diet had a different effect. Further investigation of the intestinal microbiome of cats, the effects of *Saccharomyces* in conjunction with *S. cerevisiae* derived β-1,3-1,6-glucan on the gut associated lymphoid tissue, and the overall feline immune system is needed.

Though this study was not designed to detect the differences between sex, we noticed (though not significant because of the small sample size) a difference between male and female cats. It is unclear whether there could be an immunological mechanism, more strongly associated with female cats, that could cause a rabies antibody suppression after the oral supplementation of β-glucans. Previous research showed a difference in immunological response to antigens based on sex in other species [29,30,31,32,33]. There are indications that testosterone, androgen, and estrogen levels in any species have immunological regulating capacities [34,35,36]. Repeating the study with a higher number of animals may bring clarity in this potential difference.

## 5. Conclusions

In contrast to other animal species, the β-1,3-1,6-glucan supplemented diet did not give the same effect on anti-rabies antibodies in cats. While in other animal species, the β-1,3-1,6-glucans showed significantly higher antibody titers than the non-supplemented feed, in cats, it was the inverse. The reasons for this remain unclear and need further investigation.

## Figures and Tables

**Table 1:** Epidemiological data based on age and sex.

	Group A (Placebo)	Group B (Test)
**Sex**		
Male	9 (50%)	9 (53%)
Female	9 (50%)	8 (47%)
**Age in months**		
4–11	6 (33%)	6 (35%)
12–23	8 (44%)	7 (41%)
24–35	3 (17%)	3 (18%)
36+	1 (6%)	1 (6%)

## Abbreviations

DLA	Dog Leukocyte Antigen Complex
FLA	Feline Leukocyte Antigen Complex
GALT	Gut-Associated Lymphoid Tissue
MHC	Major Histocompatibility Complex
TLR	Toll-Like Receptors
RFFIT	Rapid Florescent Foci Inhibition Test
RVNA	Rabies Virus Neutralizing Antibody

## References

[1] Gibson G., Scott K., Rastall R., Tuohy K., Hotchkiss A., Dubert-Ferrandon A., Gareau M., Murphy E.F., Saulnier D., Loh G. (2010). Dietary prebiotics: Current status and new definition. Food Sci. Technol. Bull. Funct. Foods.

[2] Hong F., Hansen R.D., Yan J., Allendorf D.J., Baran J.T., Ostroff G.R., Ross G.D. (2003). β-Glucan Functions as an Adjuvant for Monoclonal Antibody Immunotherapy by Recruiting Tumoricidal Granulocytes as Killer Cells. Am. Assoc. Cancer Res..

[3] Brown G.D., Taylor P.R., Reid D.M., Willment J.A., Williams D.L., Martinez-Pomares L., Wong S.Y.C., Gordon S. (2002). Dectin-1 is a Major β-Glucan Receptor on Macrophages. J. Exp. Med..

[4] Han B., Baruah K., Cox E., Vanrompay D., Bossier P. (2020). Structure-Functional Activity Relationship of b-Glucans From the Perspective of Immunomodulation: A Mini-Review. Front. Immunol..

[5] Netea M.G., Joosten L.A., Latz E., Mills K.H., Natoli G., Stunnenberg H.G., O’Neill L.A., Xavier R.J. (2016). Trained immunity: A program of innate immune memory in health and disease. Science.

[6] Marakalala M.J., Williams D.L., Hoving J.C., Engstad R., Netea M.G., Brown G.D. (2013). Dectin-1 plays a redundant role in the immunomodulatory activities of b-glucan-rich ligands in vivo. Microbes Infect..

[7] Vojtek B., Mojžišová J., Smrčo P., Drážovská M. (2017). Effects of orally administered β–1,3/1,6–glucan on vaccination responses and immunological parameters in dogs. Food Agric. Immunol..

[8] Hetland G., Sandven P. (2002). β-1,3-glucan reduces growth of *Mycobacterium tuberculosis* in macrophage cultures. FEMS Immunol. Med. Microbiol..

[9] Stuyven E., Cox E., Vancaeneghem S., Arnouts S., Deprez P., Goddeeris B.M. (2009). Effect of β-glucans on an ETEC infection in piglets. Vet. Immunol. Immunopathol..

[10] Vetvicka V., Vannucci L., Sima P. (2014). The Effects of β-Glucan on Pig Growth and Immunity. Open Biochem. J..

[11] Procedure. https://www.rabieselisa.com/procedure/.

[12] Rupprecht C.E., Fooks A.R., Abela-Ridder B. (2018). Laboratory Techniques in Rabies.

[13] R Core Team (2020). R: A Language and Environment for Statistical Computing.

[14] Bates D., Maechler M., Bolker B., Walker S. (2015). Fitting Linear Mixed-Effects Models Using lme4. J. Stat. Softw..

[15] Krakowski L., Krzyzanowski J., Wrona Z., Siwicki A.K. (1999). The effect of nonspecific immunostimulation of pregnant mares with 1,3/1,6 glucan and levamisole on the immunoglobulins levels in colostrum, selected indices of nonspecific cellular and humoral immunity in foals in neonatal and postnatal period. Vet. Immunol. Immunopathol..

[16] Ai Q., Mai K., Zhang L., Tan B., Zhang W., Xu W., Li H. (2007). Effects of dietary β-1 3-glucan on innate immune response of large yellow croaker, *Pseudosciaena crocea*. Fish. Shellfish Immunol..

[17] Selim K.M., Reda R.M. (2015). Beta-Glucans and Mannan Oligosaccharides Enhance Growth and Immunity in Nile Tilapia. N. Am. J. Aquac..

[18] Soltanian S., Stuyven E., Cox E., Sorgeloos P., Bossier P. (2009). Beta-glucans as immunostimulant in vertebrates and invertebrates. Crit. Rev. Microbiol..

[19] Ewaschuk J.B., Johnson I.R., Madsen K.L., Vasanthan T., Ball R., Field C.J. (2012). Barley-derived β-glucans increases gut permeability, ex vivo epithelial cell binding to *E. coli*, and naïve T-cell proportions in weanling pigs. J. Anim. Sci..

[20] Zhang M., Kim J.A., Huang A.Y.C. (2018). Optimizing Tumor Microenvironment for Cancer Immunotherapy: β-Glucan-Based Nanoparticles. Front. Immunol..

[21] Akdis M., Aab A., Altunbulakli C., Azkur K., Cota R.A., Crameri R., Duan S., Eiwegger T., Eljaszewicz A., Ferstl R. (2016). Interleukins (from IL-1 to IL-38), interferons, transforming growth factor β, and TNF-α: Receptors, functions, and roles in diseases. J. Allergy Clin. Immunol..

[22] Stuyven E., Verdonck F., Van Hoek I., Daminet S., Duchateau L., Remon J.P., Goddeeris B.M., Cox E. (2010). Oral administration of β-1,3/1,6-glucan to dogs temporally changes total and antigen specific IgA and IgM. Clin. Vaccine Immunol..

[23] Day M. (2016). Cats are not small dogs: Is there an immunological explanation for why cats are less affected by arthropod-borne disease than dogs?. Parasites Vectors.

[24] Morris K. (2009). The Feline Major Histocompatibility Complex. Univ. Syd. Undergrad. Res. J..

[25] Handl S., Dowd S.E., Garcia-Mazcorro J.F., Steiner J., Suchodolski J.S. (2011). Massive parallel 16S rRNA gene pyrosequencing reveals highly diverse fecal bacterial and fungal communities in healthy dogs and cats. FEMS Microbiol. Ecol..

[26] Tizard I.R., Jones S.W. (2018). The microbiota regulates immunity and immunologic diseases in dogs and cats. Vet. Clin. N. Am. Small Anim. Pract..

[27] Bradshaw J.W.S. (2006). The Evolutionary Basis for the Feeding Behavior of Domestic Dogs (Canis familiaris) and Cats (Felis catus). J. Nutr..

[28] Garcia-Mazcorro J.F., Ishaq S.L., Rodriguez-Herrera M.V., Garcia-Hernandez C.A., Kawas J.R., Nagaraja T.G. (2019). Review: Are there indigenous *Saccharomyces* in the digestive tract of livestock animal species? Implications for health, nutrition and productivity traits. Animal.

[29] Cohn D.A. (1979). Sensitivity to androgen. A possible factor in sex differences in the immune response. Clin. Exp. Immunol..

[30] McGraw K.J., Ardia D.R. (2005). Sex differences in carotenoid status and immune performance in zebra finches. Evol. Ecol. Res..

[31] Klein S., Flanagan K. (2016). Sex differences in immune responses. Nat. Rev. Immunol..

[32] Giefing-Kröll C., Berger P., Lepperdinger G., Grubeck-Loebenstein B. (2015). How sex and age affect immune responses, susceptibility to infections, and response to vaccination. Aging Cell.

[33] Roved J., Westerdahl H., Hasselquist D. (2017). Sex differences in immune responses: Hormonal effects, antagonistic selection, and evolutionary consequences. Horm. Behav..

[34] Ahmed S.A., Penhale W.J., Talal N. (1985). Sex Hormones, Immune Responses, and Autoimmune Diseases Mechanisms of Sex Hormone Action. Am. J. Pathol..

[35] Fish E.N. (2008). The X-files in immunity: Sex-based differences predispose immune responses. Nat. Rev. Immunol..

[36] Schneider-Hohendorf T., Görlich D., Savola P., Kelkka T., Mustjoki S., Gross C.C., Owens G.C., Klotz L., Dornmair K., Wiendl H. (2018). Sex bias in MHC I shaping of adaptive immunity. Proc. Natl. Acad. Sci. USA.

